# Evaluation and Optimization of Cement Slurry Systems for Ultra-Deep Well Cementing at 220 °C

**DOI:** 10.3390/ma17215246

**Published:** 2024-10-28

**Authors:** Zhi Zhang, Zhengqing Ai, Lvchao Yang, Yuan Zhang, Xueyu Pang, Zhongtao Yuan, Zhongfei Liu, Jinsheng Sun

**Affiliations:** 1 R&D Center for Ultra Deep Complex Reservoir Exploration and Development, CNPC, Korla 841000, China; zhangz-tlm@petrochina.com.cn (Z.Z.); azq-tlm@petrochina.com.cn (Z.A.); yanglc-tlm@petrochina.com.cn (L.Y.); yuanzt-tlm@petrochina.com.cn (Z.Y.); zhzf-tlm@petrochina.com.cn (Z.L.); 2 Engineering Research Center for Ultra-Deep Complex Reservoir Exploration and Development, Xinjiang Uygur Autonomous Region, Korla 841000, China; 3 Xinjiang Key Laboratory of Ultra-Deep Oil and Gas, Korla 841000, China; 4 Petrochina Tarim Oilfield Company, Korla 841000, China; 5 State Key Laboratory of Deep Oil and Gas, China University of Petroleum (East China), Qingdao 266580, China; z23020120@s.upc.edu.cn (Y.Z.); sunjsdri@cnpc.com.cn (J.S.); 6 School of Petroleum Engineering, China University of Petroleum (East China), Qingdao 266580, China

**Keywords:** oil well cement, additives, anti-retrogression materials, abnormal gelation, fluid loss, ultra-deep wells, strength retrogression

## Abstract

With the depletion of shallow oil and gas resources, wells are being drilled to deeper and deeper depths to find new hydrocarbon reserves. This study presents the selection and optimization process of the cement slurries to be used for the deepest well ever drilled in China, with a planned vertical depth of 11,100 m. The bottomhole circulating and static temperatures of the well were estimated to be 210 °C and 220 °C, respectively, while the bottomhole pressure was estimated to be 130 MPa. Laboratory tests simulating the bottomhole conditions were conducted to evaluate and compare the slurry formulations supplied by four different service providers. Test results indicated that the inappropriate use of a stirred fluid loss testing apparatus could lead to overdesign of the fluid loss properties of the cement slurry, which could, in turn, lead to abnormal gelation of the cement slurry during thickening time tests. The initial formulation given by different service providers could meet most of the design requirements, except for the long-term strength stability. The combined addition of crystalline silica and a reactive aluminum-bearing compound to oil well cement is critical for preventing microstructure coarsening and strength retrogression at 220 °C. Two of the finally optimized cement slurry formulations had thickening times more than 4 h, API fluid loss values less than 50 mL, sedimentation stability better than 0.02 g/cm^3^, and compressive strengths higher than 30 MPa during the curing period from 1 d to 30 d.

## 1. Introduction

The exploitation of deep and ultra-deep oil and gas resources has become a crucial strategic area for increasing reserves and production in the petroleum industry, especially in China [1,2,3,4,5,6,7]. With more and more hydrocarbon discoveries in deep formations, the depth of oil and gas well drilling is quickly increasing, reaching 10,000 m and beyond [8,9,10]. During the drilling and completion processes of these deep wells, complex geological conditions characterized by high temperatures and pressures (HTHPs) (temperature >200 °C, pressure >100 MPa) are typically encountered [1]. The temperature in extremely deep wells can reach up to 240 °C, posing severe challenges to the performance of oil well cement systems used to provide zonal isolation in these wells. On the one hand, the ultra-high temperature environment may cause the chemical bonds of the primary chemical additives in the cement slurry to break and desorb from the cement particles, leading to issues such as premature setting, fluid loss, and particle settling in the cement slurry, which could severely impact the cementing quality and may even cause complete well construction failures [11]. On the other hand, the ultra-high temperature environment could lead to microstructure coarsening, increased porosity, strength retrogression, and increased permeability of the hardened cement during a long service period, adversely affecting the integrity of the cement sheath and the lifespan of oil and gas wells [12,13,14,15,16,17].

Traditional silica-enriched oil well cement systems are preferred for high-temperature cementing applications due to their good compatibility with additives, ease of quality control, and low cost. However, recent research has indicated that only adding silica is insufficient to prevent long-term strength retrogression of oil well cement under ultra-high temperature conditions of 200 °C and above [12], and even using particle size distribution optimization strategies based on close-packing theory (by mixing different particle sizes of silica, microsilica, and nanosilica) showed limited effectiveness in preventing long-term strength retrogression [13,17]. This is primarily because such methods fail to change the composition of hydration products, and the amorphous C-S-H gel generated in these systems easily undergoes structural change or crystallization under ultra-high temperature curing environments above 200 °C, which can lead to microstructure coarsening and the retrogression of various physical and mechanical properties [12]. Studies have shown that reducing the total content of the inherently unstable C-S-H gel in the early stages of cement hydration [18] and introducing aluminum atom substitution for calcium positions in the C-S-H structure (to form a C-A-S-H gel) [16,19] can enhance the high-temperature stability of set cement. Industrial waste such as fly ash and slag that are rich in aluminum-bearing compounds have been proven to be highly effective anti-strength retrogression (ASR) materials for traditional silica-cement systems [16,19]. In contrast, highly inert materials such as rutile seem to be unreactive and ineffective, despite its high aluminum oxide content [17]. Additionally, the various aluminum-bearing anti-strength retrogression materials are often incompatible with traditional oil well cement additives (such as retarders, fluid loss additives, and dispersants) and can easily cause slurry gelation issues, which could in turn affect the workability of the cement slurry.

Chemical additives are essential materials for oil cement slurries to adjust engineering properties such as the thickening time, fluid loss, rheology, and sedimentation stability. These additives mainly include retarders, fluid loss agents, dispersants, and suspending agents [11]. Currently, the thermally most stable chemical additives are usually synthetic ternary or quaternary polymers. These synthetic polymer additives can be designed at the molecular level to exhibit specific functional properties, which has become a focus point of oil well cement research [20,21,22,23,24,25]. At temperatures below 200 °C, it is relatively easy to design and synthesize chemical additives to control the thickening time and other properties of oil well cement systems [26,27]. However, at temperatures of 200 °C and higher, chemical additives can easily undergo desorption and decomposition, losing their effectiveness [25,28,29,30]. In recent years, extensive research has resulted in a series of commercial high-temperature-resistant additives by fine-tuning their molecular structures [11]. Nevertheless, designing high-temperature-resistant cement slurry systems is still quite challenging today due to various factors such as incompatibility between the chemical additives and anti-strength retrogression materials at ultra-high temperatures, high initial slurry viscosity, abnormal gelation due to the excessive use of polymer additives, and high sensitivity to the dosage of chemical additives (especially retarders) [31,32,33,34,35]. The insufficient design of oil well cement slurries can lead to operation efficiency and safety problems during field applications, which must be addressed through the extensive optimization of cement slurry formulations and evaluation of the corresponding slurry properties.

In summary, the development of deep petroleum resources calls for well cementing systems suitable for ultra-high temperature and pressure environments. Although extensive research has been devoted to this area in recent years, previous studies have mainly focused on the optimization of one or two additives/admixtures to address specific problems associated with high-temperature cementing. Few studies have considered the compatibility of various new additives and admixtures. The main purpose of this study is to develop oil well cement systems suitable for ultra-high temperature application environments in a real-world scenario. The slurry is to be used to cement the deepest well ever drilled in China (TaKe #1 well, Tarim oilfield, China National Petroleum Corporation), with a planned vertical depth of 11,100 m. The bottomhole circulating and static temperatures of the well have been estimated to be 210 °C and 220 °C, respectively, while the bottomhole circulating pressure has been estimated to be 130 MPa. Comprehensive experimental evaluation was conducted to make sure that the slurry could meet both the field operation requirements and provide long-term zonal isolation throughout the life of the well. The following design requirements were established: (1) the thickening time should be adjustable in the range of 3 h to 8 h; (2) the initial slurry consistency at room temperature should be less than 40 Bc; (3) the API fluid loss value should be less than 50 mL; (4) the sedimentation stability as measured by the sample density differences from top to bottom should be less than 0.02 g/cm^3^; and (5) the 24 h compressive strength should be higher than 24 MPa and exhibit no strength retrogression during at least a 30 d curing. Tests were conducted according to the API standard, employing Aksu Class G cement as well as chemical additives and anti-strength retrogression materials supplied by four different service providers. By comparing the performances of different cement slurry systems, two slurries were selected for further optimization, and all properties of the finally optimized systems met the design requirements.

## 2. Materials and Experimental Routines

### 2.1. Raw Materials

**Figure 1:**
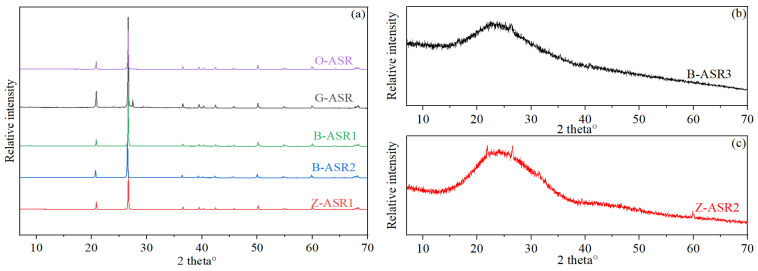
X-ray diffraction test results of various ASR materials: (**a**) O-ASR, G-ASR, B-ASR1, B-ASR-2, Z-ASR1, (**b**) B-ASR3, and (**c**) Z-ASR2.

**Figure 2:**
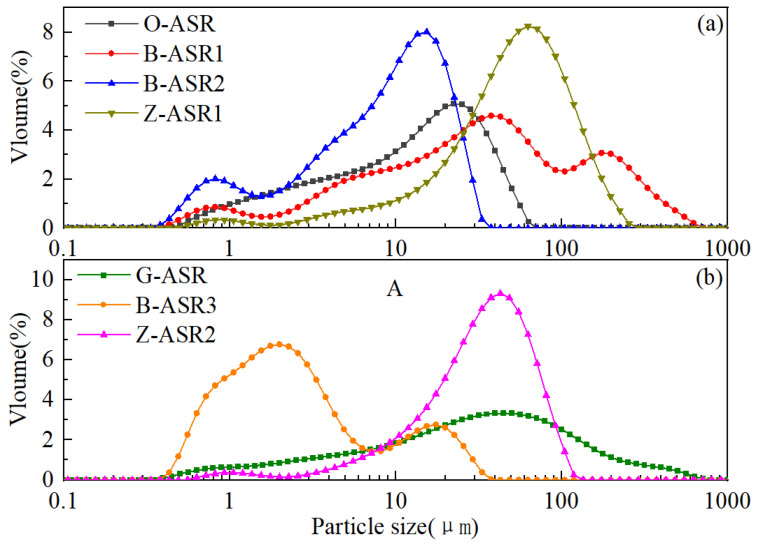
Particle size distribution analysis of various ASR materials: (**a**) O-ASR, B-ASR1, B-ASR2, Z-ASR1, and (**b**) G-ASR, B-ASR3, and Z-ASR2.

The raw materials utilized in this study included Aksu Class G oil well cement by the Aksu Cement Company (Aksu, China) as well as a variety of chemical additives and mineral admixtures (ASR materials) supplied by four different service providers for the Tarim oilfield. As shown in Table 1, each additive or admixture was given a code name in order to identify them properly. It should be noted that O-ASR, B-ASR1, B-ASR2, and Z-ASR1 are all crystalline-silica-based materials with different particle sizes, while G-ASR, B-ASR3, and Z-ASR2 are proprietary ASR materials with aluminum-containing compounds and other minerals. The mineral compositions of the various ASR materials evaluated by X-ray diffraction tests are given in Figure 1. It is obvious that O-ASR, B-ASR1, B-ASR2, and Z-ASR1 presented typical crystalline silica peaks, while G-ASR mainly presented crystalline silica peaks with an additional small unidentified peak at around 27°. In contrast, B-ASR3 and Z-ASR2 presented amorphous hump peaks ranging from 15° to 35°, which is similar to the amorphous material in fly ash and slag [16,19]. Representative oxide analysis results of the cement and ASR materials obtained by X-ray fluorescence tests [36] are given in Table 2, which further indicate that the compositions of the crystalline-silica-based ASR materials were mainly silica (purity ≥ 95%), while the compositions of B-ASR3 and Z-ASR2 apparently contained significant amounts of other compounds. O-ASR was primarily silica (89% purity) with minor amounts of other compounds, which was also consistent with XRD test results. The particle size distributions (PSD) of the cement and various anti-strength retrogression materials are illustrated in Figure 2, and the characteristic sizes are given in Table 3.

During the preliminary design stage, crystalline-silica-based ASR materials (O-ASR and G-ASR) were used to compare the performances of the cement slurries produced by using chemical additives from different service providers. The cement slurries were subsequently optimized for thickening time, sedimentation stability, strength, permeability and fluid loss properties. Seven slurries were tested for thickening time optimization; eight slurries were evaluated for sedimentation stability optimization; eight slurries were tested for strength and permeability evaluation; and eight slurries were tested for fluid loss property optimization. It was found that high dosages of fluid loss additives could cause the abnormal gelation of cement slurries, and seven slurries were tested to investigate such phenomenon. The relatively poor physical and mechanical property test results obtained during the preliminary design stage calls for the inclusion of non-silica-based ASR materials and corresponding chemical additives. Two slurry systems with the best performances were selected for further optimization, and the testing results of the two final slurries are presented.

### 2.2. Slurry and Set Cement Preparation

The preparation and testing of the cement slurries followed standard procedures (API 10B-2 [37], GB/T 19139-2012 [38]) with slight modifications when necessary. The specific slurry formulations were gradually optimized based on the test results obtained, which are given in the test results section. The preparation of set cement samples consisted of the following steps. (1) Pour cement slurries into stainless-steel mold (25 mm × 70 mm) and puddle with a glass rod to remove entrained air. (2) Place the filled molds inside an HTHP autoclave for curing. (3) Increase the temperature/pressure of the autoclave to the target condition of 220 °C/50 MPa. (4) After the designated curing time is reached, decrease the temperature/pressure of the autoclave to room condition. (5) For permeability and compressive strength analysis, cut samples into 25 mm × 50 mm cylinders. (6) For sedimentation analysis, cut samples into four 25 mm × 16 mm disks and mark them as top, mid-1, mid-2, and bottom, from top to bottom of the 25 mm × 70 mm cylinder.

## 3. Testing Equipment and Methods

### 3.1. Thickening Time Test

The thickening time test of the cement slurry was conducted using a HTHP consistometer following standard API procedures. During a typical thickening time test, the cement slurry was stirred in a slurry cup at a constant speed of 150 RPM. The resistance encountered by the stirring paddle inside the slurry cup was measured and converted to a consistency parameter measured in Bearden consistency (Bc) units. Temperature and pressure were ramped to 210 °C and 130 MPa, respectively, within a period of 2 h.

### 3.2. Fluid Loss Test

The fluid loss test was conducted using a HTHP stirred fluid loss testing apparatus manufactured by Liaoning Bestride Petroleum Equipment Manufacturing Co. Ltd. (Shenyang, China) (model BSRD-7071F) according to the API standards. The test was conducted at 210 °C with an inlet pressure of 7 MPa and a back pressure of approximately 0.5 MPa (to prevent the filtrate from boiling and vaporizing). The fluid loss value was calculated using Equation (1) if no gas blow through occurred and Equation (2) if gas blow through occurred [37]:

(1) $$V_{API}=2V_{30}$$

(2) $$V_{cal}=2V_{t}\sqrt{\frac{30}{t}}$$

where *V*_*API*_ and *V*_*cal*_ represent the API fluid loss and the calculated fluid loss after gas blow through, respectively. *V*_30_ is the volume of liquid collected within 30 min, *V*_*t*_ is the volume of liquid collected when gas blow through occurs, and *t* is the time of gas blow through. As will be discussed later in this study, fluid loss tests conducted according to the API standard “Performing a static fluid-loss test using stirred fluid-loss apparatus” failed due to the extremely high temperatures, and modifications to the test methods had to be introduced.

### 3.3. Sedimentation Stability Test

The sedimentation stability of the cement slurry was assessed based on the maximum density difference of a set cylindrical cement sample with a diameter of 25 mm and a height of 70 mm. The sample was cut into four equal sections (as described in Section 2.2) with their densities measured according to Archimedes’ principle (i.e., by measuring the mass of the sample in the air and the mass of the sample when suspended in water). More detailed procedures can be found in API standard RP10B-2 [37].

### 3.4. Mechanical Strength Test

The determination of compressive strength was conducted by employing a universal testing machine, which was outfitted with a video extensometer to facilitate the measurement of sample deformation and strain. Three replicate specimens were tested under each condition to derive both the mean and standard deviation of the compressive strength test results. The loading rate applied during testing was maintained at 0.3 mm/min.

### 3.5. Water Permeability Test

The permeability of set cement was assessed using water as the testing medium. The inlet pressure, regulated by an automatic piston pump, was set at 2 MPa, while the outlet pressure remained at atmospheric levels; the confining pressure was maintained at 5 MPa. The permeability value was calculated using Darcy’s law. To ensure statistical significance, two duplicate specimens were tested to derive the mean results.

### 3.6. Mercury Intrusion Porosimetry Test

The pore size of set cement was analyzed using a Quantachrome mercury intrusion pore size analyzer (model PM 33) manufactured by Quantachrome Instruments, Boynton Beach, FL, USA. Samples prepared for the analysis had a total mass of approximately 1 g and consisted of small pieces of hardened cement with their largest dimensions less than 5 mm. The analysis was conducted under a maximum pressure of 32,000 psi (220 MPa).

### 3.7. Thermogravimetry Analysis

The decomposition behavior of both the chemical additive and hydration products of cement was evaluated using the thermogravimetry analysis (TGA) method. A thermogravimetry instrument manufactured by Setaram, Lyon, France (model Setline STA) was employed. Tests were conducted in alumina crucibles with nitrogen as protective gas. For liquid chemical additives, the samples were first dried in an oven at 105 °C to constant weight; for solid chemical additives, the samples were directly used. TGA tests on chemical additives were conducted by ramping the temperature from 25 °C to 600 °C at a rate of 10 °C/min. For set cement, the samples were vacuum dried for 3 days and manually ground using an agate mortar. TGA tests on set cement were conducted by first heating the samples at 105 °C for 1 h to remove all evaporable water in hydration products; final test data obtained by temperature ramping from 105 °C to 1000 °C at a rate of 5 °C/min were used.

### 3.8. X-Ray Diffraction Analysis

The mineral composition of the set cement was analyzed using the X-ray diffraction (XRD) analysis method. The sample preparation method was the same as that used for the TGA tests. A diffractometer manufactured by Malvern Panalytical (Almelo, Netherlands) (model Aeris) was utilized. The diffractometer was equipped with a 600 W Cu-anode source operated at 40 kV and 15 mA. Scans were conducted within an angular range of 7° to 70° (2θ angle), employing a 0.01° 2θ step size with an exposure time of 75.22 s per step. The sample was backloaded to the sample holder to minimize the impact of preferred orientation.

## 4. Comparison of Cementing Systems of Different Service Providers

### 4.1. TGA Test Results of Cement Additives

**Figure 3:**
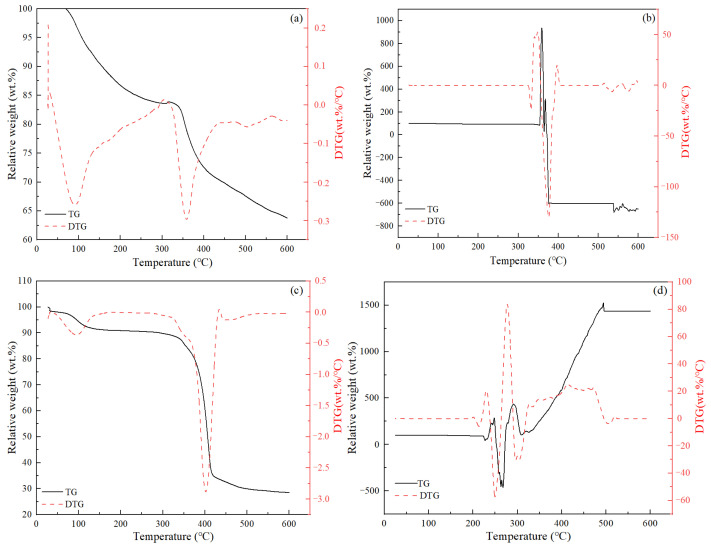
Representative TGA test results of the cement additives: (**a**) O-FR1; (**b**) B-FL; (**c**) O-FR2; (**d**) B-RT.

TGA tests were employed to evaluate the decomposition temperature of the various chemical additives employed in this study. Representative test results are given in Figure 3. It can be seen that the decomposition behavior of various additives varied widely. Most additives exhibited gradual reductions in weight with increasing temperature, and the decomposition process typically consisted of multiple stages, which were reflected as the appearance of multiple peaks in the DTG curves (see Figure 3a,c). In several special cases, the weight reduction was not obvious until a critical temperature was reached, and then the sample weight was observed to fluctuate dramatically (see Figure 3b,d), sometimes even exceeding its original weight (Figure 3d). In these special cases, the samples were observed to have expanded significantly after the test. Table 4 lists the critical temperature values corresponding to the decomposition peaks for the normal tests as well as those corresponding to the initial fluctuation points for the special cases. These values were considered as the critical failure temperature of these additives in a dried state. It was clear that for the additives with multiple decomposition peaks, the first peaks appeared at temperatures less than 200 °C, while the second or third peaks appeared at temperatures significantly higher than 200 °C. For additives with only one decomposition peak (or critical failure temperature), the values were all higher than 200 °C.

### 4.2. Cement Slurry Thickening Time Test Results of Preliminary Designs

**Figure 4:**
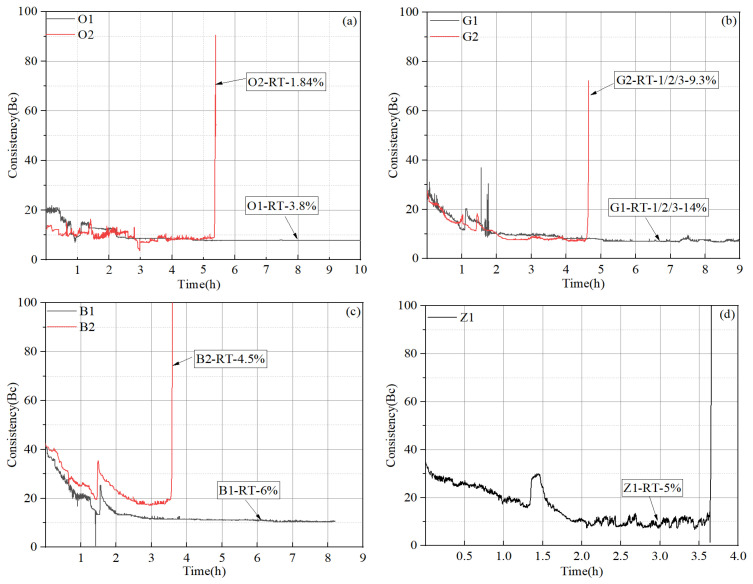
Thickening time test results of the cement slurries from different service providers: (**a**) slurries O1 and O2; (**b**) slurries G1 and G2; (**c**) slurries B1 and B2; (**d**) slurry Z1.

The thickening test results and corresponding formulation designs for the cement slurries are provided in Figure 4 and Table 5. It can be seen from Figure 4 that the thickening time is adjustable by changing the dosages of retarders, and “right-angle setting” (consistency curve jumps straight up at time of setting) behavior is common at such high temperature conditions (220 °C). The initial consistency values of all four systems were relatively low (ranging from 12 Bc to 40 Bc), suggesting that these slurries should not be difficult to pump in field applications. In most systems, only one retarder was needed to adjust the thickening time, while three retarders (G-RT-1, G-RT-2, and G-RT-3) were used in slurry “G” systems. Clearly, the amounts of retarder needed to achieve the desirable thickening time varied significantly among the different systems. Retarder O-RT appeared to exhibit the highest efficiency and could achieve a thickening time of more than 5 h in slurry “O” systems with a dosage of only 1.84%, while the retarders in slurry “G” systems appeared to have the lowest efficiency, and a total dosage of 9.3% was needed to obtain a thickening time of approximately 4.6 h. Additionally, it is clear that in most systems, there was a small jump in slurry consistency at approximately 1.5 h, which is generally associated with the abnormal gelation of the cement slurry. Nevertheless, in all of these cases, the abnormal gelation was relatively insignificant.

### 4.3. Set Cement Sedimentation Test Results of Preliminary Designs

**Figure 5:**
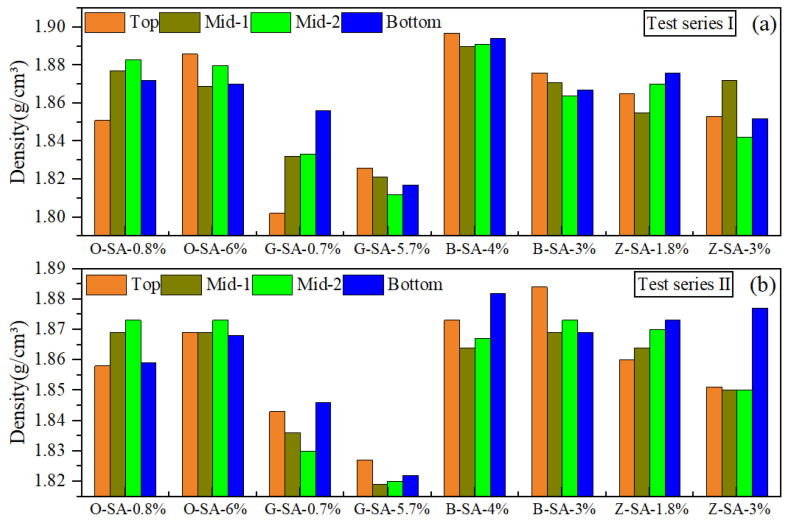
Sedimentation test results of different systems: (**a**) test series I; (**b**) test series II.

The slurry formulation designs obtained after thickening time optimization (i.e., slurries O2, G2, B2, and Z1 in Table 5) were further employed as the base slurry to evaluate the influence of suspension aid on the sedimentation stability of these different systems. Each system was tested with two different dosages of suspension aid, and the corresponding sedimentation test results are shown in Figure 5. Two replicate samples were made for each dosage of suspension aid (designated as test series I and II, respectively) to check the repeatability of the test results. It can be seen that within the range studied, a vast majority of the samples exhibited excellent sedimentation stability with the maximum density differences from the top to bottom sections less than 0.02 g/cm^3^, indicating excellent sedimentation stability control of all of the suspension aids employed. The average final density of most systems was approximately 1.87 g/cm^3^, except for slurry “G” systems, which had a density of approximately 1.82–1.83 g/cm^3^.

### 4.4. Set Cement Physical and Mechanical Property Test Results of Preliminary Designs

**Figure 6:**
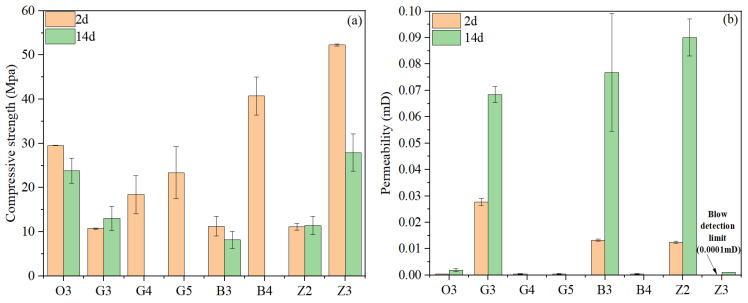
Set cement property evolution with time for preliminary design slurries: (**a**) compressive strength; (**b**) permeability.

After optimization for sedimentation stability, several slurry designs were obtained for an evaluation of the physical and mechanical properties of hardened cement, as shown in Table 6. All slurries were used to produce 2-day cure samples, while only selected slurries were used to produce 14-day cure samples to study the strength stability over time. The compressive strength and permeability test results of these slurries are shown in Figure 6. It was observed that the 2-day compressive strengths of three slurries containing 40% anti-strength retrogression material G-ASR (slurries G3, B3, Z2) were very low (approximately 10 MPa), suggesting that they would not meet the design requirement. Additionally, while the strength of these three slurries were relatively stable during further curing until 14 days, the permeability of the set cement experienced significant increases, which were all above 0.05 mD after a 14-day cure, also missing the design requirements. When the dosage of G-ASR was increased from 40% to 60%, the 2-day compressive strength of slurry G4 increased significantly to 18 MPa. When G-ASR was replaced with O-ASR with finer particle sizes and higher silica purity, the 2-day compressive strength of slurry G5 further increased to 23 MPa. Similarly, by replacing 40% G-ASR with 60% O-ASR in slurry B3, the compressive strength of slurry B4 increased to 40 MPa. These observations were consistent with previous studies in that adding a higher dosage and/or finer particle size of silica typically leads to a higher compressive strength of the oil well cement, especially in the early curing period [13,17]. However, as demonstrated in many previous studies [12,17,39], only adding silica to oil well cement systems could not prevent the long-term strength retrogression of oil well cement systems. Indeed, strength reductions and permeability increases could be observed in both slurry O3 and slurry Z3, therefore, additional anti-strength retrogression material would be needed to stabilize their long-term strength.

### 4.5. Cement Slurry Fluid Loss Test Results of Preliminary Designs

**Figure 7:**
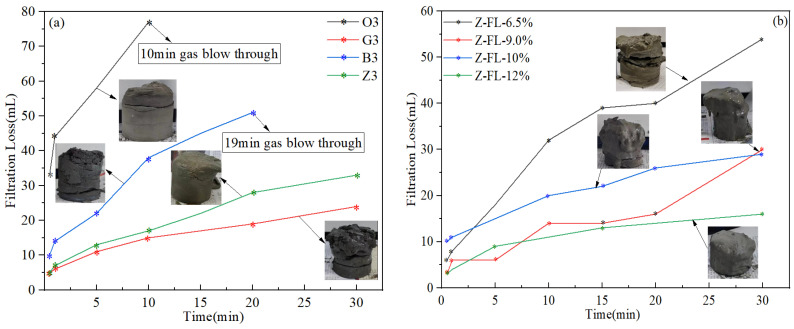
Fluid loss test results of various cement slurries: (**a**) slurries O3, G3, B3, and Z3; (**b**) slurry Z systems with different dosages of Z-FL.

According to the API standard, when conducting a static fluid loss test using a stirred fluid loss test apparatus, the cement slurry should be stirred during the heating stage, plus an additional 30 min at the final temperature, before the device is inverted to measure the filtrate. However, due to the extremely high temperature of this study, the cement slurry would have completely “dried out” at the end of the stirring stage when the test was conducted according to the standard API procedures, causing the cement to bind to the stirring shaft and completely losing its flowability. This is because the stirred fluid loss test apparatus generally uses nitrogen gas as the pressurizing medium, and the pressure applied during the stirring stage is typically 3.5 MPa. Since the density of water is only 0.854 g/cm^3^ at 210 °C/3.5 MPa, the cement slurry may experience severe sedimentation and water evaporation at such high-temperature and low-pressure conditions. Therefore, a slight modification to the test method was introduced: the cement slurry was stirred during the heating stage until the temperature reached 150 °C, the test device was then inverted and the stirring was stopped, then the temperature was further increased to 210 °C, and the filtrate was collected after the temperature stabilized. The fluid loss test results are shown in Figure 7, which show the amounts of filtrate collected as a function of time. Slurry O3 and B3 all experienced gas blow through within the 30-min testing period and the thickness of the filter cake collected reached nearly 8 cm (which was essentially the height of the entire sample after the loss of fluid), suggesting extremely poor fluid loss control performances. Although slurry G3 had low fluid loss values, the thickness of its filter cake also reached 8 cm. Among the four different systems evaluated, slurry Z2 exhibited the best performance in controlling fluid loss, with API fluid loss value of 66 mL and a filter cake thickness of 6 cm. Moreover, as shown in Figure 7b, with further increases in the dosage of fluid loss additive, the amount of filtration loss of the slurry Z system could be greatly reduced. When the dosage was increased to 9–12%, the API fluid loss was close to or lower than the design requirement of 50 mL, noting that the API fluid loss was two times the measured filtration loss according to Equation (1).

### 4.6. Abnormal Gelation of Cement Slurries

**Figure 8:**
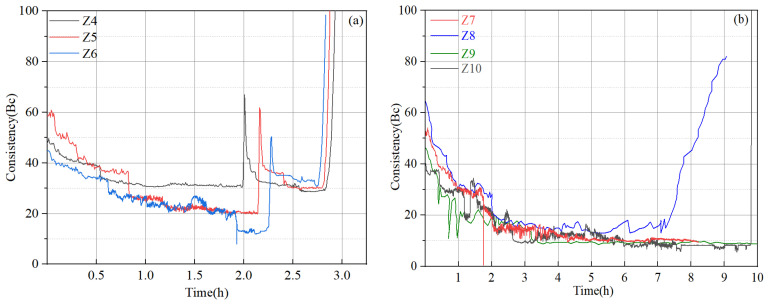
Thickening time test results of slurry Z system: (**a**) slurries Z4, Z5, and Z6; (**b**) slurries Z7, Z8, Z9, and Z10.

**Figure 9:**
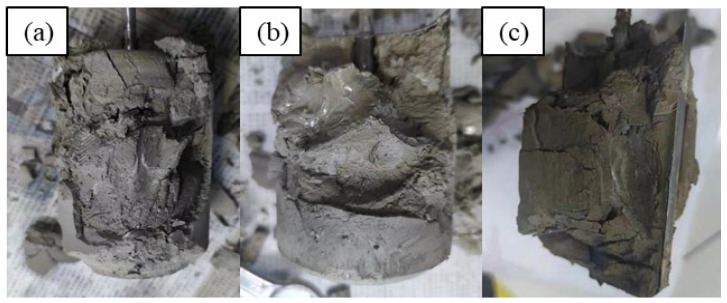
Abnormal gelation behavior of slurries: (**a**) Z4; (**b**) Z5, and (**c**) Z6.

Apparently, in order to meet the fluid loss design requirement, high dosages of fluid loss additive are needed, according to the test results in Section 4.5. Thickening time tests were conducted based on several new slurry designs (Table 7), and representative test results are given in Figure 8. The addition of a high dosage of a fluid loss additive significantly increased the viscosity of the cement slurries, with the initial consistency ranging between 40 Bc and 60 Bc, suggesting that these slurries may be difficult to pump in field applications. In several of these designs (slurries Z4, Z5, Z6), abnormal gelation phenomena were observed: the consistency evolution curves experienced dramatic and sudden jumps followed by sudden drops with equal or smaller magnitudes before the slurries finally thickened. When these tests were stopped and the samples were taken out, it was observed that the cement was set but did not harden (Figure 9), suggesting that abnormal gelation led to false setting. By slightly adjusting the dosages of the retarder, fluid loss additive, and friction reducer, the abnormal gelation issue could be effectively mitigated. However, the test results seemed to be highly sensitive to small changes in additive dosage, and it was almost impossible to determine what combinations of additive were the optimum design. This will also cause problems in field applications, where it is difficult to precisely control the additive dosages.

## 5. Evaluation of Finally Optimized Cementing Systems

### 5.1. Formulation Optimization and Cement Slurry Properties of Final Designs

**Figure 10:**
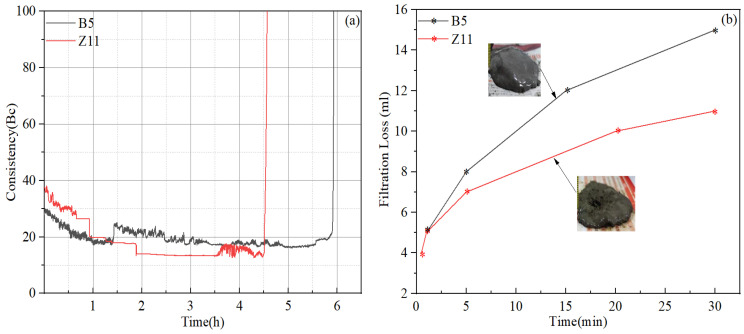
Test results of the finally optimized cement slurries: (**a**) thickening time; (**b**) fluid loss.

By comparing the test results of the cement systems provided by four different service providers, it can be concluded that the sedimentation stability and thickening time performances of all systems could meet the design requirements. However, the fluid loss and long-term strength stability of almost all systems need further optimization. During this section, the slurry “B” and slurry “Z” systems were selected for further optimization by introducing new additives and adjusting the dosage of existing ones. The finally optimized slurry designs are shown in Table 8, and the thickening time test curves are shown in Figure 10.

For the fluid loss test, a further modification to the test method was introduced, which consisted of the following steps. (1) The cement slurry was conditioned in a HTHP consistometer at 210 °C/130 MPa for 30 min to simulate downhole environments, and the temperature and pressure ramping rates employed were the same as those used for the thickening time tests. (2) After conditioning, the temperature and pressure of the consistometer were decreased to 90 °C and atmospheric pressure to allow the slurry to be transferred to the stirred fluid loss test apparatus, which was preheated to 90 °C. (3) The stirred fluid-loss cell was inverted, and the temperature was increased to 210 °C and stabilized. (4) The filtrate was collected and measured. This test method is similar to that described in the API standard “Performing static fluid-loss test using non-stirred fluid-loss cell”. Therefore, the stirred fluid-loss cell was effectively used as a non-stirred fluid-loss cell in this modified test method. As shown in Figure 10, the fluid loss measured using this newly modified test method was significantly less than that measured using the previous test method discussed in Section 4.5, despite equal or lower dosages of fluid loss additives. It can be inferred that the stirred fluid-loss test apparatus is not suitable for conditioning slurries at ultra-high temperatures due to its low pressure capability, and could significantly overestimate the fluid loss values.

### 5.2. Set Cement Physical and Mechanical Property Test Results of Final Designs

**Figure 11:**
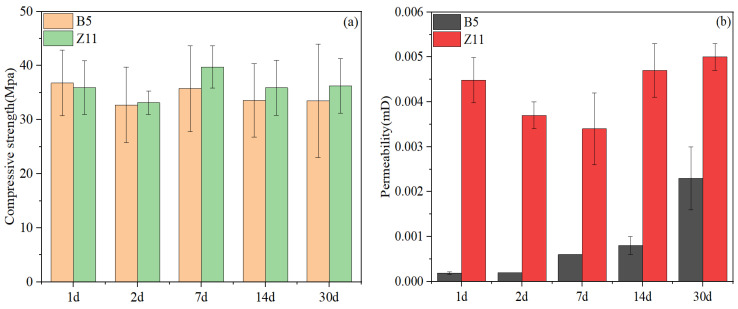
Set cement property evolution with time for final design slurries: (**a**) compressive strength; (**b**) permeability.

According to previous studies, reactive aluminum-containing compounds are critical to control the long-term strength stability of silica-enriched oil well cement systems. Therefore, in the final two slurry designs, combinations of two types of ASR admixtures were added to the oil well cement systems, one being crystalline silica (B-ASR1, B-ASR2, and Z-ASR1), and the other being newly developed alumina-based admixtures (B-ASR3, Z-ASR2). The compressive strength and permeability test results of the two finally optimized cement systems are shown in Figure 11. It can be seen from Figure 11a that the compressive strengths of slurries B5 and Z11 were almost identical, with their differences well within the experimental errors. Additionally, the compressive strength exhibited strong stability over the 30-d curing period, which varied within a very narrow range between 33 MPa and 40 MPa. Figure 11b suggests that slurry Z11 had a higher permeability than slurry B5, especially during the early curing period. However, the permeability of slurry Z11 exhibited a much better stability that that of slurry B5 during the curing period from 2 d to 30 d, with very little increase over the 30 d curing period.

### 5.3. Set Cement Microstructure and Composition Analysis of Final Designs

**Figure 12:**
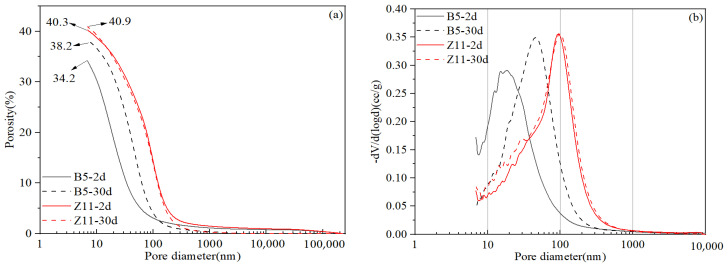
MIP pore size analysis results of set cement after different curing times: (**a**) cumulative distribution; (**b**) frequency distribution.

The macroscopic physical and mechanical properties of oil well systems are closely related to their microstructure. It has been demonstrated in several previous studies that microstructure coarsening is the cause of the high-temperature strength retrogression of oil well cement systems [12,15,16]. In general, the compressive strength decline will only occur when the microstructure coarsening has reached a certain threshold. It takes a much longer time to observe strength declines, while microstructure changes can be observed during the early curing period. Therefore, microstructure analysis can serve as a more effective early-warning indicator regarding whether a system is stable or not at high curing temperatures. Figure 12 shows the MIP test results of the two final cement slurry systems cured for different periods. It can be seen that slurry B5 had a finer initial microstructure with a porosity of 34.2% and a median pore size of 20 nm after being cured for 2 d. However, the microstructure experienced some degree of coarsening during further curing from 2 d to 30 d, with the porosity and median pore size increasing to 38.2% and 45 nm, respectively. In contrast, slurry Z11 had a coarser initial microstructure, with a porosity of 40.3% and a median pore size of 100 nm after being cured for 2 d, which almost did not change during further curing from 2 d to 30 d. The microstructure test results presented good agreement with the permeability test results shown in Figure 11.

**Figure 13:**
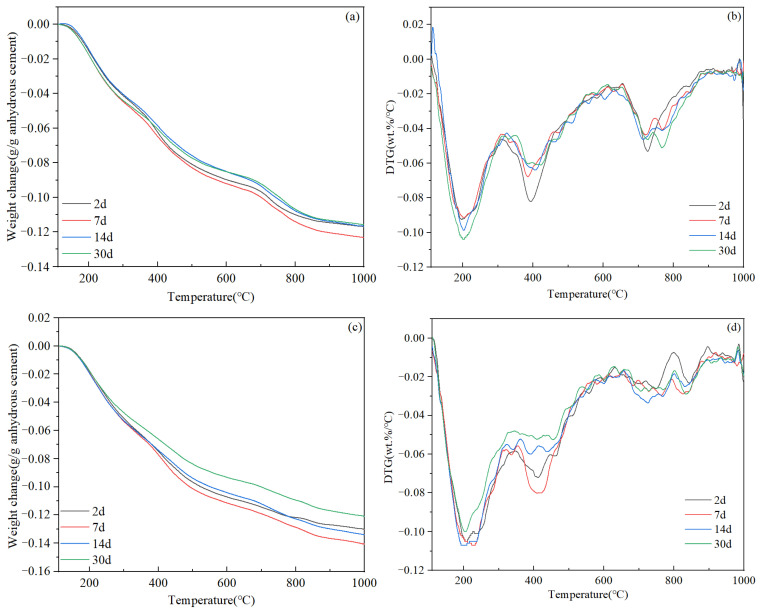
TGA test results of set cement after different curing times: (**a**) thermogravimetry and (**b**) derivative thermogravimetry curves of slurry B5; (**c**) thermogravimetry and (**d**) derivative thermogravimetry curves of slurry Z11.

The TGA analysis results are displayed in Figure 13. From 2 d to 30 d of curing, the measured weight loss due to the loss of chemically combined water (in hydration products) for slurry B5 and slurry Z11 remained relatively stable at 0.11–0.13 g/g anhydrous cement and 0.12–0.14 g/g anhydrous cement, respectively. Three primary decomposition peaks could be observed in the slurry B5 samples. The decomposition peak at approximately 200 °C can be attributed to the loss of water in semi-crystalline C-(A)-S-H and tombermorite phases; the decomposition peak at approximately 400 °C can be attributed to the further loss of water in semi-crystalline C-(A)-S-H and aluminum-containing compounds such as calcium silicoaluminate hydrate, katoite, and grossular [40]; and the decomposition peak at approximately 760 °C can be primarily attributed to the loss of water in xonotlite. It can be seen from Figure 13b that all decomposition peaks remained relatively stable over time, suggesting that no significant changes of mineral phases occurred during this curing period. In contrast, samples of Slurry Z11 exhibited two primary decomposition peaks at 200 °C and 400 °C and some weak peaks between 630 °C and 850 °C, suggesting that these samples contained little to no xonotlite phase. Additionally, the decomposition peak at 400 °C appeared to decrease slightly with increasing curing time in both the B5 and Z11 samples, which may be associated with some unknown structural change in the C-(A)-S-H phase during long-term curing.

**Figure 14:**
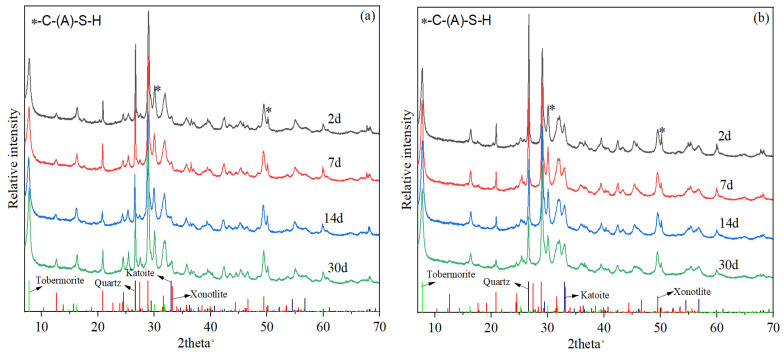
XRD test results of set cement after different curing times: (**a**) slurry B5; (**b**) slurry Z11.

The X-ray diffraction (XRD) test results of the slurries at different curing times are presented in Figure 14. The unreacted quartz peaks were obvious in both slurries at all curing times. The main hydration products of slurries B5 and Z11 both included semi-crystalline C-(A)-S-H (hump peak in the range of 28.5° to 33.5°), tobermorite, and an alumina-bearing phase such as katoite and grossular. Compared to traditional silica-enriched well cement systems with no aluminum-bearing ASR materials [12,13,14], the tobermorite peak at 7.9° in these two slurries was significantly enhanced, which is critical for long-term strength stabilization [16,19]. The presence of a xonotlite phase in the slurry B5 samples were confirmed by the unique peaks at 12.7°, which were almost absent in slurry Z11 samples. The katoite and grossular peaks at 32.9° and 56.6° in slurry Z11 were more significant, possibly due to the higher dosage of Z-ASR2 as well as its higher alumina content compared to B-ASR3 (See Table 2). Semi-crystalline C-(A)-S-H is known to be a key phase contributing to the early strength of the cement, while the conversion of C-S-H to xonotlite is known to cause strength retrogression during long-term curing at ultra-high temperatures [12,14]. However, although the early formation of a significant xonotlite phase typically leads to relatively low early strength of the high-temperature-cured cement, it can significantly enhance the long-term strength stability due to stronger phase stability [18]. During the curing period from 2 d to 30 d, the XRD profiles of slurries B5 and Z11 exhibited no significant change, indicating that the crystallization of semi-crystalline C-(A)-S-H into xonotlite was possibly inhibited by the combined addition of multiple ASR materials.

## 6. Conclusions

Developing high-temperature resistant cementing systems is highly critical for the construction of ultra-deep wells to tap into deep petroleum resources. The main purpose of this study was to screen and optimize cement slurry designs for the final casing string of the first 10,000-m well in China, using more than 30 additives and admixtures from different service providers. A comprehensive experimental program was conducted to investigate the cementing fluid property at the simulated circulating temperature of 210 °C as well as the set cement properties at the simulated static temperature of 220 °C.

It was found that most of the commercial cement additives manufactured by various service providers had good temperature resistance, with their main decomposition temperatures above 200 °C, based on the TGA test results. The thickening time of various cement slurry systems was adjustable by changing the retarder dosage. However, the efficiency of different types of retarders varied significantly, and hence the total amount of retarders needed to reach the desirable thickening time varied widely from 1.84% to 9.3%. The use of crystalline silica alone is apparently not able to prevent the strength retrogression of oil well cement systems at a 220 °C curing temperature. The combined use of two types of anti-strength retrogression (ASR) materials (one being silica-based and the other being reactive alumina-based) is critical to stabilize the long-term strength of oil well cement. However, the optimal dosages of various ASR materials need further investigation.

By comparing two standard test methods for evaluating the fluid loss properties of oil well cement slurries, in other words, (1) performing a static fluid-loss test using stirred fluid-loss apparatus, and (2) performing a static fluid-loss test using non-stirred fluid-loss apparatus. It was determined that method (1) is not suitable for ultra-high temperature testing due to the low pressure applied during the conditioning stage. Further testing is needed to determine the upper temperature limit for such test methods.

## Figures and Tables

**Table 1:** Code names for the additives and admixtures by different service providers.

Additive	Supplier O	Supplier G	Supplier B	Supplier Z
Anti-strength retrogression material	O-ASR	G-ASR	B-ASR1, B-ASR2, B-ASR3	Z-ASR1, Z-ASR2
Retarder	O-RT	G-RT1, G-RT2, G-RT3	B-RT	Z-RT1, Z-RT2
Suspension aid	O-SA	G-SA	B-SA	Z-SA1, Z-SA2, Z-SA3
Fluid loss additive	O-FL	G-FL	B-FL	Z-FL
Friction reducer	O-FR1, O-FR2		B-FR	Z-FR1, Z-FR2
Reinforcing agent			B-RFA	Z-RFA1, Z-RFA2
	O-DFM		B-DFM	Z-DFM
Elastomer				Z-ELS

**Table 2:** Oxide analysis results by X-ray fluorescence tests (wt.%).

Oxide Name	G-ASR	B-ASR3	Z-ASR2	B-ASR1	B-ASR2	Cement
CaO	2.87	9.58	15.45	1.45	0.019	65.69
SiO_2_	89.29	60.98	46.89	95.41	96.32	19.18
Fe_2_O_3_	0.66	6.53	0.26	0.87	0.06	5.69
SO_3_	0.24	0.41	0.21	0.21	0.08	3.35
Al_2_O_3_	3.03	14.52	18.75	1.03	1.31	2.96
MgO	0.95	1.19	0.59	-	0.06	1.75
K_2_O	1.91	1.89	0.05	0.06	0.15	0.65
Na_2_O	0.30	1.08	9.27	0.19	0.94	0.19

**Table 3:** Summary of the key parameters of the particle size distribution of various bulk materials.

Parameter	Cement	O- ASR	G- ASR	B- ASR1	B- ASR2	B- ASR3	Z- ZSR1	Z- ASR2
D(10) μm	1.04	1.74	2.47	3.72	1.15	0.782	12.5	9.66
D(50) μm	15.39	12.49	28.65	33.6	2.20	2.20	53.1	34.10
D(90) μm	48.05	33.73	143.05	217	15.5	15.50	124	70.40

**Table 4:** Critical decomposition temperature of various cement additives by TGA tests.

Additive	Temp. ^(1)^ (°C)	Additive	Temp. ^(1)^ (°C)	Additive	Temp. ^(1)^ (°C)	Additive	Temp. ^(1)^ (°C)
O-RT	80/330	G-RT1	231	B-RT	215	Z-RT	338
O-SA	114/240/464	G-RT2	286	B-SA	89/331	Z-SA	84/290
O-FL	283	G-RT3	407	B-FL	221	Z-FL	352
O-FR1	95/360	G-SA	94/288	B-FR	98/386	Z-FR	167/358
O-FR2	400	G-FL	336			Z-RFA	450
		G-FR	116/354				

^(1)^ Critical decomposition temperature.

**Table 5:** Cement slurry formulations employed during thickening time tests.

Comp. ^(1)^	O1	O2	Comp. ^(1)^	G1	G2	Comp. ^(1)^	B1	B2	Comp. ^(1)^	Z1
Cement	100	100	Cement	100	100	Cement	100	100	Cement	100
O-ASR	60	60	G-ASR	40	40	G-ASR	40	40	G-ASR	40
O-RT	3.8	1.84	G-RT1	7	4.7	B-RT	6	4.5	Z-RT	5
O-SA	0.8	0.8	G-SA	0.7	0.7	B-SA	4	4	Z-SA	1.8
O-FL	6.5	6.5	G-FL	4.2	4.2	B-FL	6	6	Z-FL	5
O-FR1	1	1	G-RT2	5.6	3.7	B-FR	5.5	5.5	Z-FR	1
O-FR2	2	2	G-RT3	1.4	0.9	-	-	-	Z-RFA	3
O-AGM	0.5	0.5	-	-	-	-	-	-	-	-
O-DFM	0.1	0.1	Z-DFM	0.5	0.5	Z-DFM	0.5	0.5	Z-DFM	0.5
Water	53.47	53.47	Water	44.5	49	Water	43	44	Water	50.4
TT ^(2)^	>750 ^(3)^	335		>539 ^(3)^	278		>493 ^(3)^	215		219

^(1)^ Slurry composition; ^(2)^ Thickening time; ^(3)^ Tests were stopped before the slurry’s final thickening time.

**Table 6:** Cement slurry formulations employed during the physical and mechanical property tests.

Comp. ^(1)^	O3	Comp. ^(1)^	G3	G4	G5	Comp. ^(1)^	B3	B4	Comp. ^(1)^	Z2	Z3
Cement	100	Cement	100	100	100	Cement	100	100	Cement	100	100
O-ASR	60	G-ASR	40	60	0	G-ASR	40	0	G-ASR	40	0
O-RT	1.8	O-ASR	0	0	60	O-ASR	0	60	O-ASR	0	60
O-SA	3	G-RT1	4.7	7.5	5	B-RT	6	4.5	Z-RT1	5	6
O-FL	6.5	G-RT2	3.7	6	4	B-SA	4	3	Z-SA1	2	2
O-FR1	1	G-RT3	0.9	1.5	1	B-FL	6	6	Z-FL	5	5
O-FR2	2	G-SA	3	1.5	1.5	B-FR	5.5	5.5	Z-FR1	1	1
O-AGM	0.5	G-FL	4.2	5	5	-	-		Z-RFA1	3	3
O-DFM	0.1	Z-DFM	0.5	1	1	Z-DFM	0.5	1	Z-DFM	0.5	1
Water	53.5	Water	44.5	43.5	49.8	Water	44	51.2	Water	49.4	57.3

^(1)^ Slurry composition.

**Table 7:** Slurry Z system formulation designs after fluid loss property optimization.

Slurry Composition	Slurry No.						
	Z4	Z5	Z6	Z7	Z8	Z9	Z10
Cement	100	100	100	100	100	100	100
O-ASR	60	60	60	60	60	60	60
Z-RT	4.5	4.5	4	6	5	4.7	4.5
Z-SA	2	2	2	2	2	2	2
Z-FL	12	12	10	12	12	10	10
Z-FR	1	1.5	2	1	1.5	2	2
Z-RFA	5	5	3	5	5	3	3
Z-DFM	1	1	1	1	1	1	1
Water	52.95	52.6	53.8	51.9	51.95	53.3	53.4
TT ^(1)^	175	172	170	>495 ^(2)^	546	>570 ^(2)^	588

^(1)^ Thickening time; ^(2)^ Tests were stopped before the slurry’s final thickening time.

**Table 8:** Cement slurry formulation design after final optimization.

Composition	Z11	Composition	B5
Cement	100	Cement	100
Z-ASR1 (silica)	40	B-ASR1 (silica)	30
Z-ASR2	35	B-ASR2 (silica)	30
Z-RT2	4	B-ASR3	17.5
Z-SA2 (L58)	3	B-RT	4.1
Z-SA3 (DRK-4L)	4	B-SA	2.3
Z-FL	4.5	B-FL	6
Z-FR1	2.4	B-FR	5
Z-FR2	0.8	B-RFA	1.13
Z-RFA1	4	B-DFM	0.1
(XWY)Z-RFA2	2	Water	59.3
Z-ELS	2		
Z-DFM	1		
Water	65		

## Data Availability

The original contributions presented in the study are included in the article, further inquiries can be directed to the corresponding author.
